# Study on the mechanism of Jiawei Shoutai Pill in the treatment of diminished ovarian reserve based on network pharmacology

**DOI:** 10.1097/MD.0000000000041729

**Published:** 2025-02-28

**Authors:** Xiaoyue Lyu, Chun Shen, Yumin Fang, Ying Zhao

**Affiliations:** a Guangzhou University of Chinese Medicine, Guangzhou, Guangdong Province, PR China; b The First Affiliated Hospital, Guangzhou University of Chinese Medicine, Guangzhou, Guangdong Province, PR China.

**Keywords:** diminished ovarian reserve, Jiawei Shoutai Pill, therapeutic target

## Abstract

Jiawei Shoutai Pill is a traditional Chinese medicine formulation used clinically by physicians to treat diminished ovarian reserve (DOR) with positive outcomes. This study aimed to explore the potential pharmacological mechanisms of Jiawei Shoutai Pill in treating DOR by using network pharmacology methods. The effective compounds from traditional Chinese medicine were collected from the TCMSP, SYMmap, and PubChem, and the corresponding targets were retrieved from PubChem, Swiss Target Prediction, and DrugBank. Additionally, targets for DOR were obtained from GeneCards and Online Mendelian Inheritance in Man databases. Subsequently, multiple networks were constructed and gene enrichment analysis was performed using Cytoscape3.9.0 software. Molecular docking and molecular dynamics simulations were conducted based on previous research results. After screening, 72 active compounds and 292 target genes of Jiawei Shoutai Pill (excluding duplicate target genes) were identified, resulting in 1371 target genes related to the disease. A total of 149 cross-target genes were identified between the drug and disease targets. Kyoto encyclopedia of genes and genomes and gene ontology analyses emphasized the various gene functions and signaling pathways involved in treating DOR. Further molecular docking and dynamics simulations partially confirmed the practicality of the action Jiawei Shoutai Pill in vivo. The pharmacological effect of the Jiawei Shoutai Pill on DOR may be related to the PI3K–AKT, TNF, and lipopolysaccharide pathways. This study paves the way for further research on the mechanism of action of the Jiawei Shoutai Pill.

## 1. Introduction

Diminished ovarian reserve (DOR) is a common reproductive endocrine disorder in women of childbearing age. It refers to the decreased capacity of the ovarian cortex to reserve oocytes in women from menarche to 40 years of age, leading to a reduction in the number and/or quality of oocytes as well as a decrease in the levels of anti-Müllerian hormone, a decrease in antral follicle count, and an increase in serum follicle-stimulating hormone, ultimately resulting in reduced potential for female fertility.^[1]^ Its clinical symptoms are mostly manifested as reduced menstrual flow, shortened menstrual cycle, abnormal uterine bleeding, amenorrhea, and infertility.^[2]^ A diminished ovarian reserve can cause dysfunction of the neuroendocrine system and the hypothalamic–pituitary–ovarian axis, affecting the corresponding neuroendocrine centers and target organs. It can also lead to various menopausal symptoms of varying degrees, such as hot flashes, night sweats, dizziness, tinnitus, irritability, anxiety, depression, sleep disorders, and a variety of complex physical and mental symptoms. Furthermore, it may induce cardiovascular and cerebrovascular diseases, fractures, neurodegenerative diseases, osteoporosis, and other conditions, posing a serious threat to the quality of life of women of childbearing age and their families.^[3]^

Currently, the treatment of DOR mainly involves hormone replacement therapy, which aims to rapidly supplement exogenous hormones to restore menstrual status and improve menopausal symptoms. However, this approach cannot improve ovulation function and may pose potential risks to estrogen–target organs such as the uterus and breasts, limiting its clinical application.^[4]^ Complementary and Alternative Medicine refers to a diverse set of healthcare systems, practices, and products that are generally not considered a part of traditional medicine, including herbal medicine formulations. Previous experimental research and clinical experience has shown that herbal medicine formulations have significant clinical efficacy.^[5]^

The Jiawei Shoutai Pill is a combination of the Shoutai Wan and Si Junzi Tang. The Shoutai Wan, which originates from Zhang Xichun’s A Record of Integrating Western Medicine into Traditional Chinese Medicine, was originally formulated to treat recurrent miscarriage. It is composed of 4 main ingredients: Cuscutae Semen (Tu Si Zi), Taxilli Herba (Sang Ji Sheng), Dipsaci Radix (Xu Duan), and Asini Corii Colla (The donkey-hide gelatin). Professor Luo Songping, the academic successor to the nationally renowned gynecologist Professor Luo Yuankai and a leading figure in traditional Chinese medicine, has combined the Shoutai Wan with Si Junzi Tang—a traditional formula known for its qi-tonifying properties and comprising Codonopsis Radix (Dang Shen), Poria (Fu Ling), Atractylodis Macrocephalae Rhizoma (Bai Zhu), and Glycyrrhizae Radix Praeparata (Zhi Gan Cao)—to create the Jiawei Shoutai Pill. Based on her extensive clinical experience, this enhanced formula is specifically designed for the treatment of diminished ovarian reserve.

Recent clinical studies have shown that Shou Tai Wan combined with qi-supplementing drugs can improve ovarian blood supply and endometrial receptivity,^[6,7]^ leading some researchers to speculate that it may have a protective effect on ovarian function. Several physicians have used Si Jun Zi Tang with modifications to treat DOR and have achieved significant therapeutic effects.^[8,9]^ Therefore, the use of modified Shou Tai Wan combined with Si Jun Zi Tang as a new strategy for treating DOR is promising. However, the pharmacological mechanisms and molecular targets require further investigation.

Based on the characteristics of traditional Chinese medicine, network pharmacology can analyze the relationships between different components of Chinese medicine, study the biological pathway signals of diseases, and establish the overall relationship between active ingredients and targets, and diseases, which is similar to the overall view of traditional Chinese medicine treatment. Currently, network pharmacology is widely used to screen the active ingredients of drugs, predict specific drug action mechanisms, analyze the main targets of active ingredients, and develop combination drugs. As a research approach, network pharmacology can also be used to explain the compatibility rules of traditional Chinese medicine and explore new indications for traditional Chinese medicine. This study applied network pharmacology, molecular docking technology, and molecular dynamics simulation technology to explore the mechanism of Jiawei Shoutai Pill in treating DOR, providing insights for the clinical treatment of DOR.

## 2. Materials and methods

### 2.1. Target acquisition

#### 2.1.1. Screening of effective components and target of Jiawei Shoutai Pill

Using TCMSP database to search “Tu Si Zi,” “Sang Ji sheng,” “Dang Shen,” “Fu Ling,” “Bai Zhu” and “Zhi Gan Cao” respectively and screen out the chemical components and corresponding targets of each Chinese medicine that simultaneously meet the criteria of oral bioavailability ≥30% and drug-likeness ≥0.18. After obtaining the effective components, the MOL.ID number was used to search for the target action of a single ingredient. Compound Excel data table was downloaded from Uniprot database, the data was optimized by “TRIM” function, and the target gene name was matched by “VLOOKU” function. Finally, the relevant target proteins of the chemical constituents obtained by the above method were annotated using the Uniprot database, and ineffective components without targets were removed.

The donkey-hide gelatin and Xuduan components were collected using the SYMmap database (http://www.symmap.org/), and the screening criteria were set to oral bioavailability value ≥ 30%. The active ingredients and SMILES were obtained from PubChem database. SMILES was imported into the Swiss Target Prediction (http://www.swisstargetprediction.ch) database to predict effective targets.

#### 2.1.2. Prediction of disease targets

With “Diminished ovarian reserve” as the key word, the search was set to score ≥10, and Genecards (https://www.genecards.org/), and OMIM (https://omim.org/) databases were used to screen for DOR disease-related targets. The target of active compound in Jiawei Shoutai Pill was intersected with DOR disease target by Venny (https://bioinfogp.cnb.csic.es/tools/venny/) and the potential key targets of Jiawei Shoutai Pill for the treatment of DOR were obtained.

### 2.2. Construction and analysis of network model

#### 2.2.1. Traditional Chinese medicine ingredients–potential targets–diseases

Cytoscape3.9.0 software was used to construct the network of “Chinese herbal ingredients–potential targets–diseases,” and CytoNCA plug-in was used for analysis. The top 5 key chemical ingredients were selected according to their degree value.

#### 2.2.2. Protein–protein interaction network (PPI)

The protein–protein interaction relationship was obtained by importing the intersection gene through the String platform (https://string-db.org/), setting the object as Homo sapiens, taking the highest confidence level of 0.900, and hiding the free gene nodes. The results were imported into Cytoscape3.9.0 software, and the network topology parameters were obtained by selecting “network Analyzer.” The downloaded TSV file was imported into Cytoscape software to construct PPI diagram, and the top 10 core targets were screened according to the degree value.

### 2.3. GO and KEGG functional enrichment analysis

The gene ontology (GO) and Kyoto encyclopedia of genes and genomes (KEGG) functional enrichment analysis of the core targets was performed by “ClusterProfiler,” “Stringin” and “Pathview” packages of R software, and visualized by MicroBioSign platform.

### 2.4. Composition–target molecular docking

Molecular docking was performed between small and large molecules with the top topological parameters. The AlphaFold (https://alphafold.ebi.ac.uk/) database was used to obtain and establish the crystal structure of the proteins, and the small molecule library of the TCMSP database was used to search for key chemical components. The protein crystal structure was dehydrated and hydrotreated using AutoDockTools1.2, and the receptor structure was prepared. The Open Babel and AutoDock programs were used to split the small molecule library, and the AutoDockTools1.2 program was used for docking. Finally, the results were imported into PyMol to visualize the docking results.

### 2.5. Molecular dynamics simulation

The “Desmond” module of Schrodinger 2019 software was used for operation. The SPC water model was used to simulate water molecules with OPLS2005 force field. To neutralize the system charge, a suitable amount of chloride ion/sodium ion was added to balance the system charge and was placed randomly in the solvated system. After the solvation system was built, the energy of the system was minimized using the default protocol integrated with the Desmond module (using the OPLS 2005 force field parameters). Temperature and pressure were maintained at 300 K and 1 atmosphere using nose-hoover temperature coupling and isotropic scale; after which a 100 ns NPT simulation was run, and the trajectory was saved at 100 ps intervals.

## 3. Results

### 3.1. Acquisition of effective active ingredients and potential targets of Jiawei Shoutai Pill

**Figure 1.**
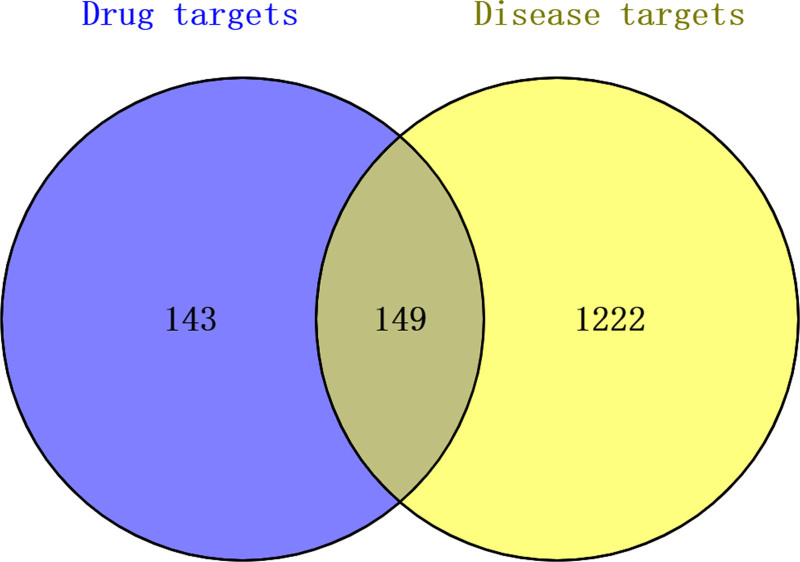
Intersection Venn diagram of Jiawei Shoutai Pill and DOR disease targets. DOR = diminished ovarian reserve.

A total of 72 effective components of the Jiawei Shoutai Pill were screened, corresponding to 292 targets (after removing duplicate values). After inputting screening conditions into the GeneCards and OMIM databases, 1371 DOR-related targets were obtained. In addition, 149 potential targets were obtained by the intersection of the targets of the Jiawei Shoutai Pill and DOR disease targets, as shown in Figure 1.

### 3.2. Construction of “traditional Chinese medicine ingredients–potential targets–disease” network

**Figure 2.**
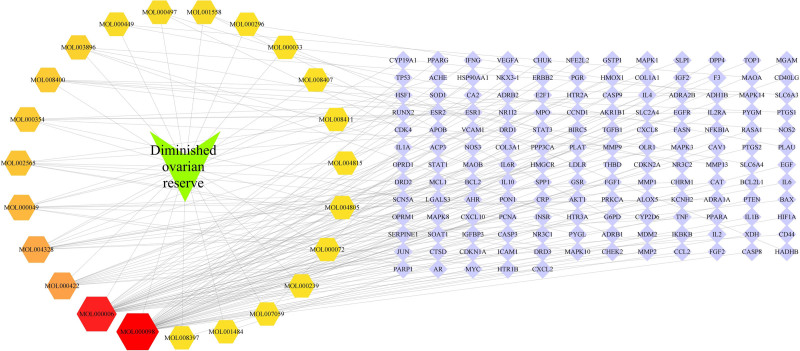
“Traditional Chinese medicine ingredients–potential targets–disease” network.

A total of 23 active ingredients and 149 potential targets of TCM compounds were visualized by Cytoscape3.9.0 software, and the TCM ingredients–potential targets–disease network was constructed, as shown in Figure 2. The network consists of 173 nodes and 172 edges. The top 5 compounds were quercetin, luteolin, kaempferol, naringenin, and 3β-acetoxyatractylone. These 5 components were speculated to be the key chemical constituents in the treatment of DOR with Jiawei Shoutai Pill.

### 3.3. Construction of PPI network

**Figure 3.**
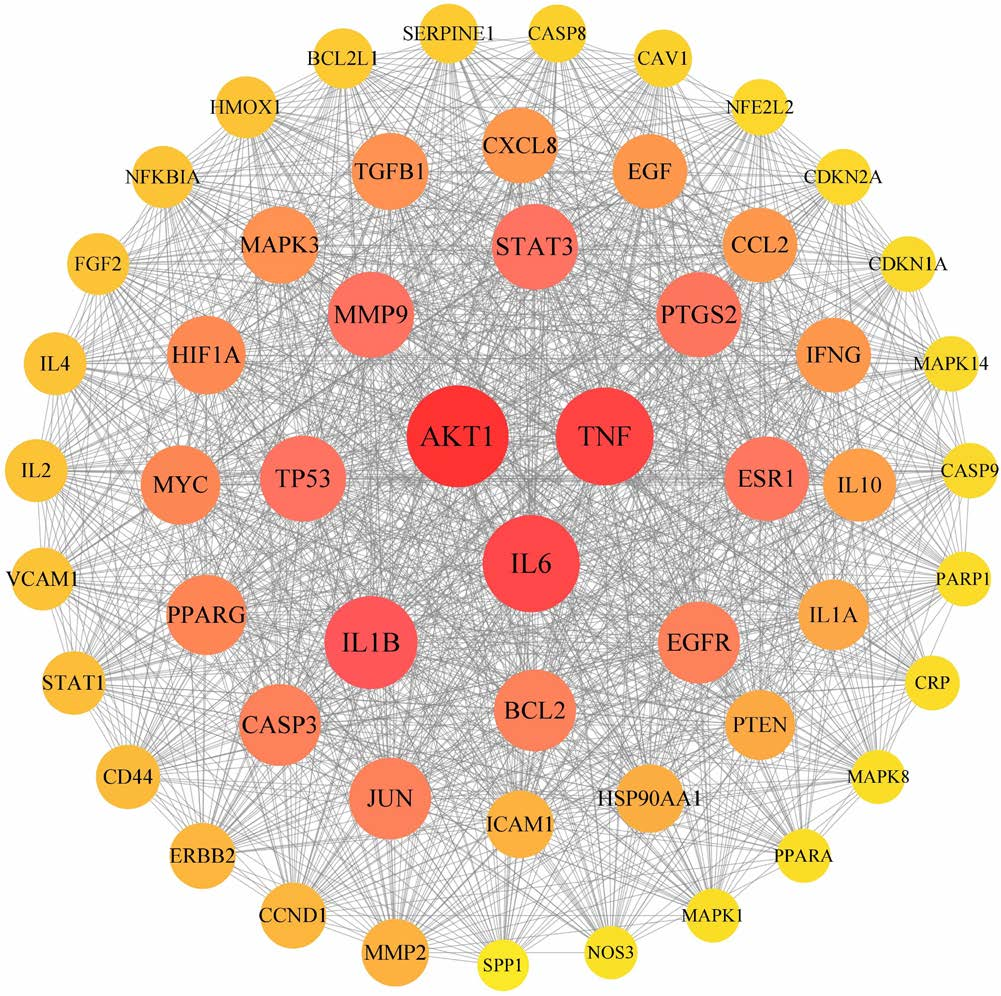
Protein–protein interaction network of Jiawei Shoutai Pill for the treatment of DOR-related targets. DOR = diminished ovarian reserve.

**Figure 4.**
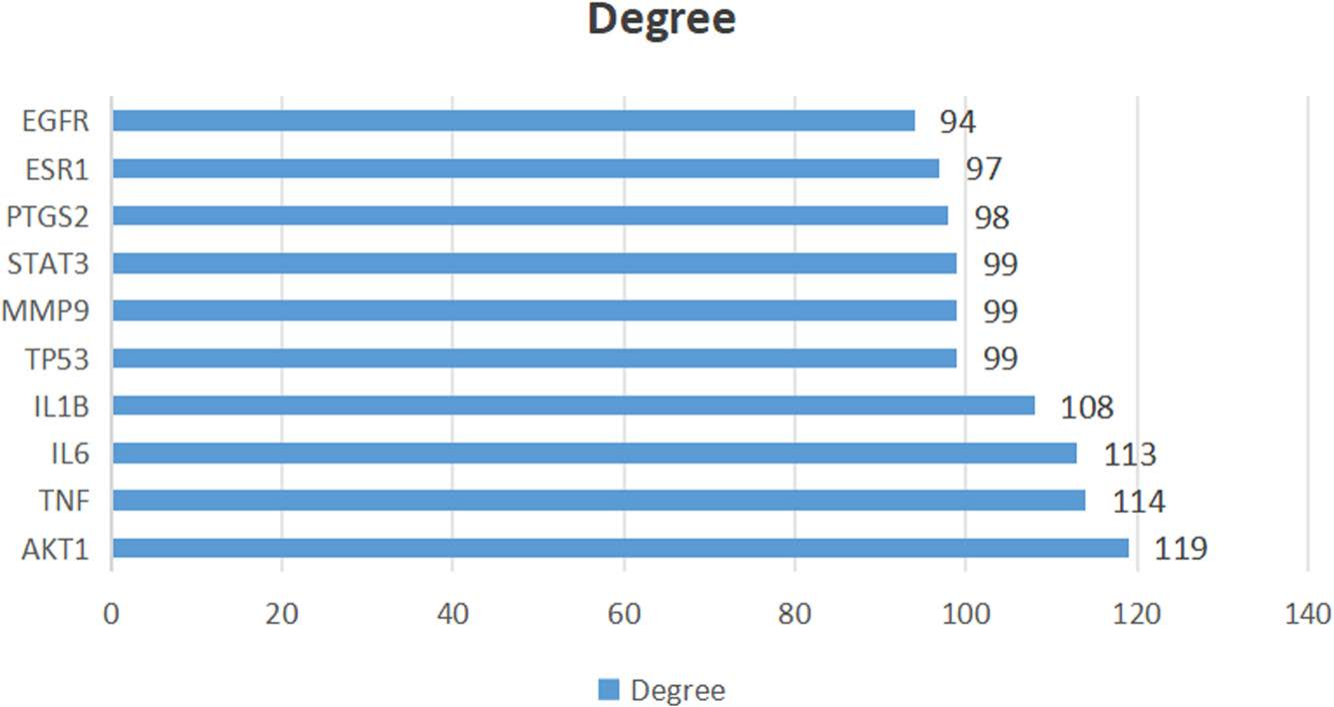
The degree value of potential targets of Jiawei Shoutai Pill in the treatment of DOR. DOR = diminished ovarian reserve.

A total of 149 potential targets were imported into the String database for PPI network construction. As shown in Figure 3, this network involved 54 nodes and 1312 edges. Nodes represent target genes, whereas edges represent interactions between the target genes. According to the degree order, the top 5 targets were protein kinase B (AKT1), tumor necrosis factor (TNF), interleukin-6 (IL-6), interleukin-1β (IL-1β), and tumor protein p53 (TP53) (Fig. 4). These targets can be considered as key targets for the treatment of DOR with the Jiawei Shoutai Pill.

### 3.4. GO and KEGG enrichment analysis

**Figure 5.**
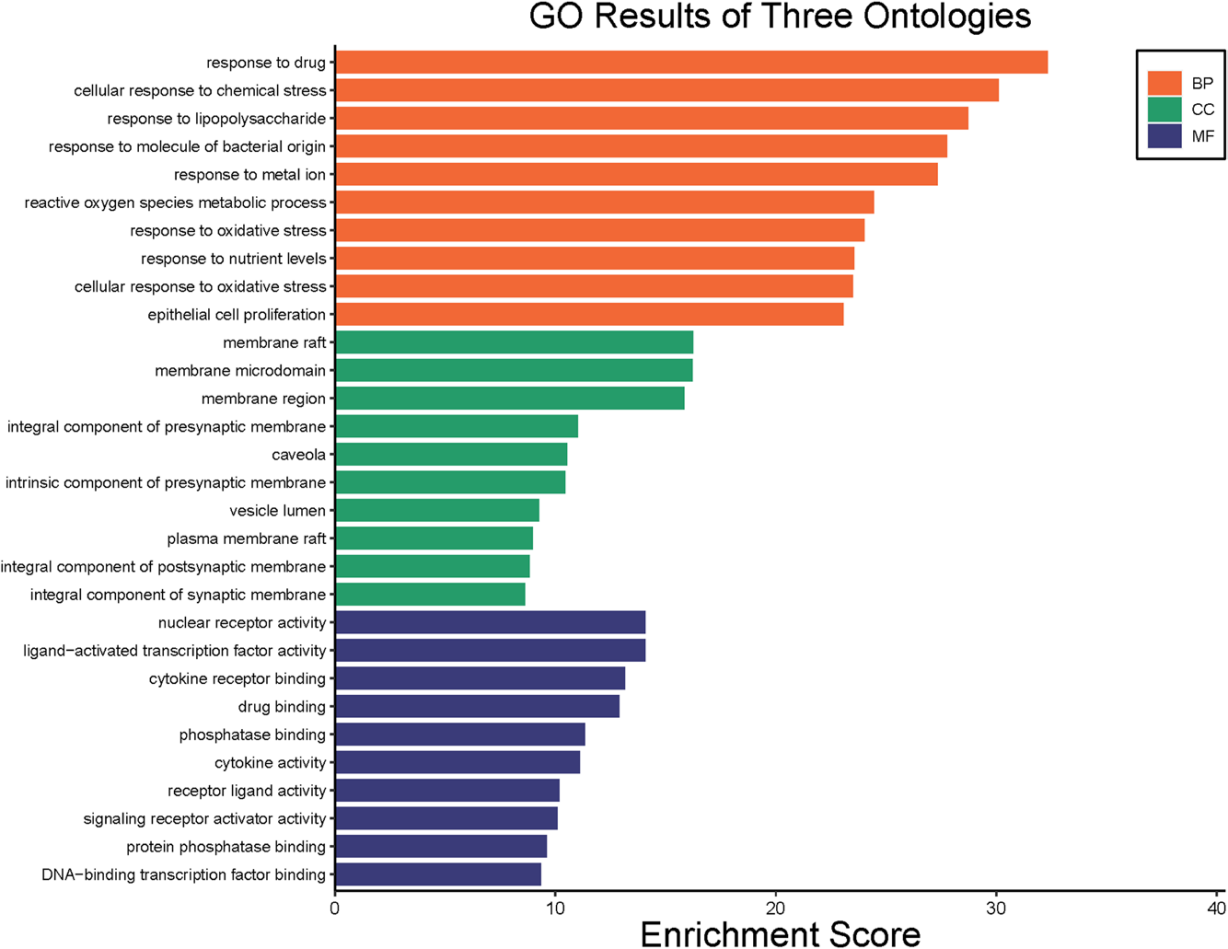
GO function enrichment analysis of potential target for Jiawei Shoutai Pill in treating. GO = gene ontology.

**Figure 6.**
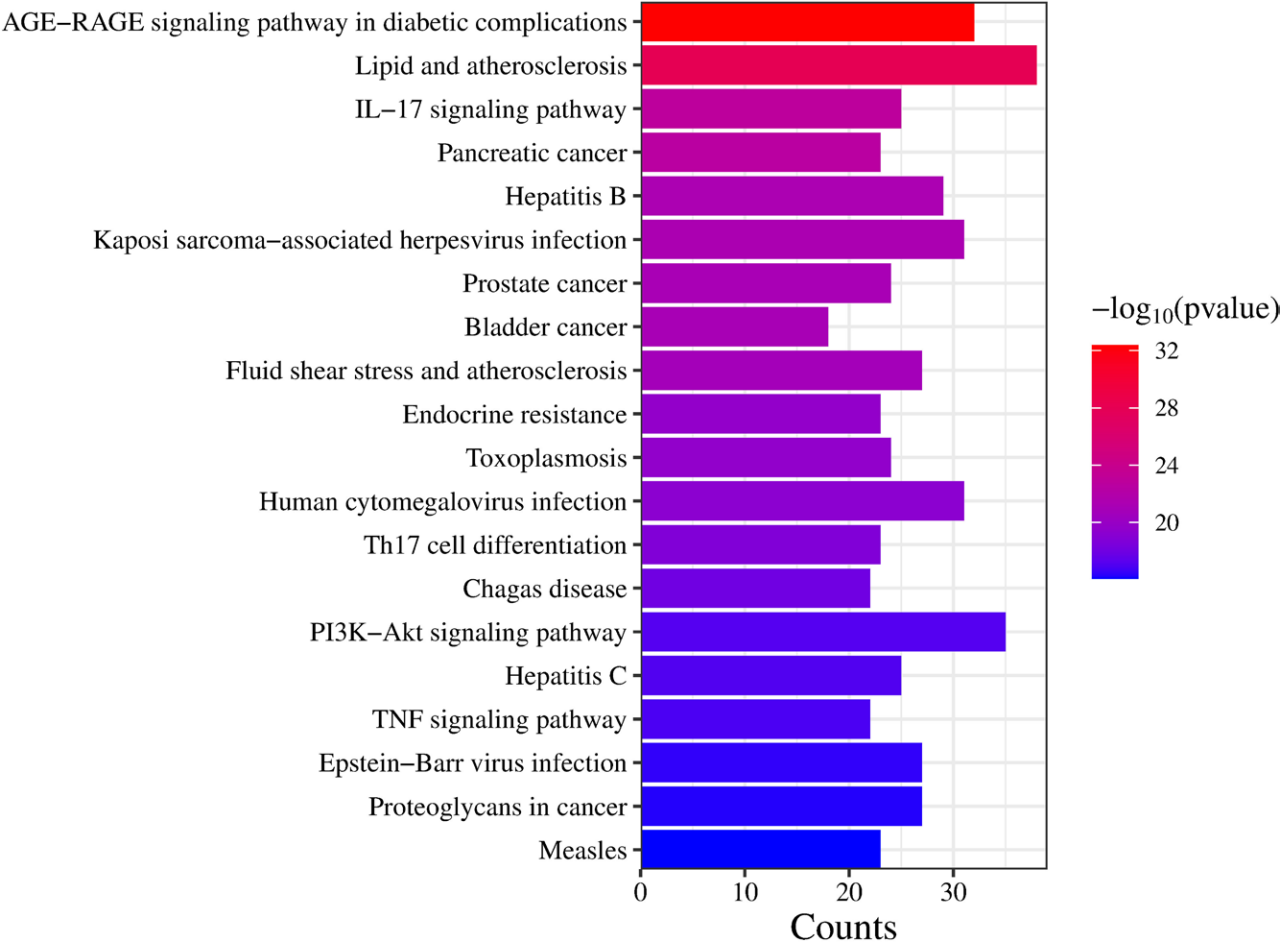
KEGG histogram of Jiawei Shoutai Pill in treatment of DOR. DOR = diminished ovarian reserve, KEGG = Kyoto encyclopedia of genes and genomes.

Through GO and KEGG gene function enrichment analyses, we elucidated the key signaling pathways involved in the treatment of DOR with Jiawei Shoutai Pill. Using a screening criterion of *P* < .05, we obtained 120 cellular components, 2646 biological processes, and 172 molecular functions. The top 10 GO entries for each category are plotted in a bar graph in Figure 5. Taking *P* < .05 as the screening condition, a total of 170 signal pathways were obtained, and the top 20 pathways were created into a histogram, as shown in Figure 6. The results showed that Jiawei Shoutai Pill could treat DOR through reactive oxygen species metabolism, oxidative stress, lipopolysaccharide metabolism and other related biological processes, as well as the phosphoinositide 3-kinase (PI3K)–AKT, TNF, lipid, and atherosclerosis signaling pathways.

### 3.5. Molecular docking results

**Figure 7.**
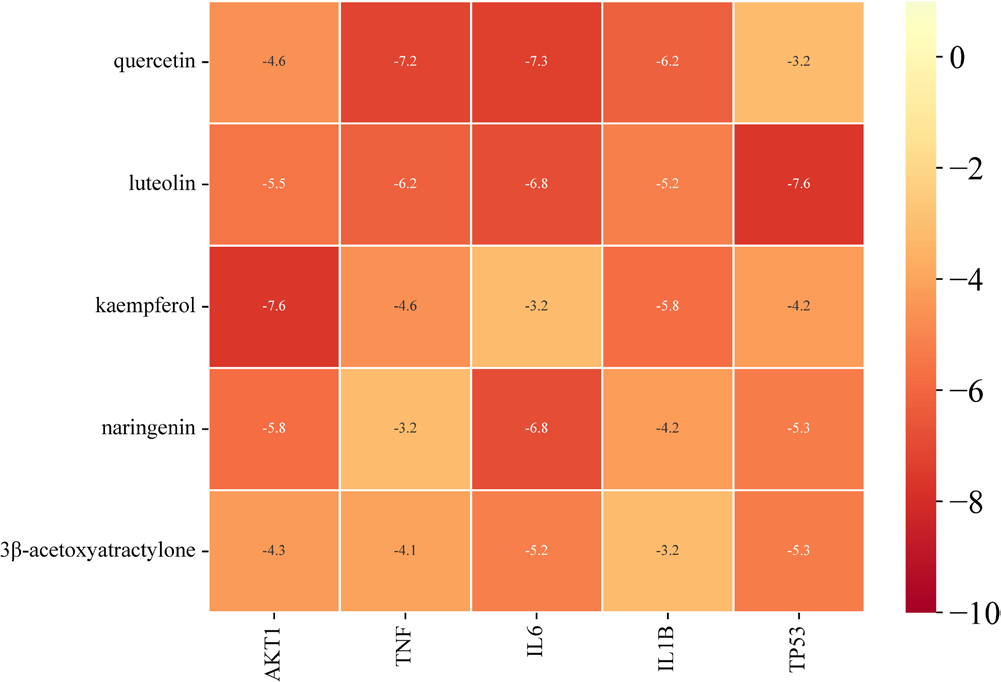
Minimum binding energy of potential active ingredients for Jiawei Shoutai Pill and key targets for treating DOR. DOR = diminished ovarian reserve.

**Figure 8.**
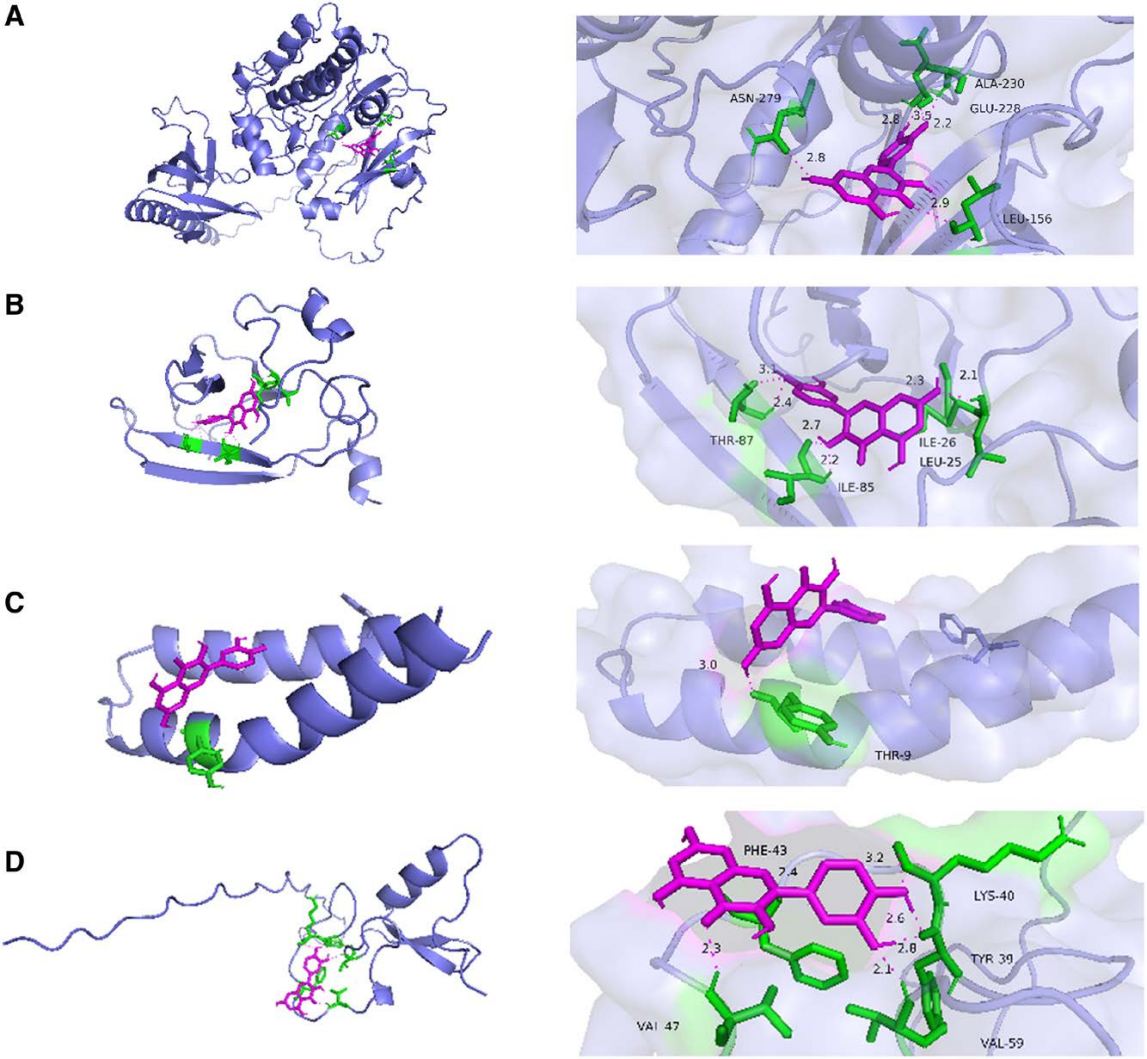
Molecular docking model of potential active ingredients for Jiawei Shoutai Pill and key targets for treating DOR. DOR = diminished ovarian reserve.

In the treatment of DOR with Jiawei Shoutai Pill, the components with relatively high degree values among the potential active ingredients included quercetin, luteolin, kaempferol, naringenin, and 3β-acetoxyatractylone. At the same time, the degree values of AKT1, TNF, IL-6, IL-1β, and TP53 target nodes were among the top in the connectivity sequence. AutodockTools1.5.6 software was used to docking the top active components with the target. The binding heat energy of molecular docking <‐1 kcal/mol indicated binding activity, and <‐5 kcal/mol indicated good binding activity, the molecular docking results are shown in Figure 7. It can be found from the figure that quercetin has good binding activities with TNF and IL-6, luteolin, 3β-acetoxyatractylone and TP53, keampferol and AKT1, naringenin and IL-6. The molecular docking simulations of keampferol–AKT1, luteolin–TP53, quercetin–IL-6, and quercetin–TNF are shown in Figure 8.

### 3.6. Molecular dynamics simulation results

**Figure 9.**
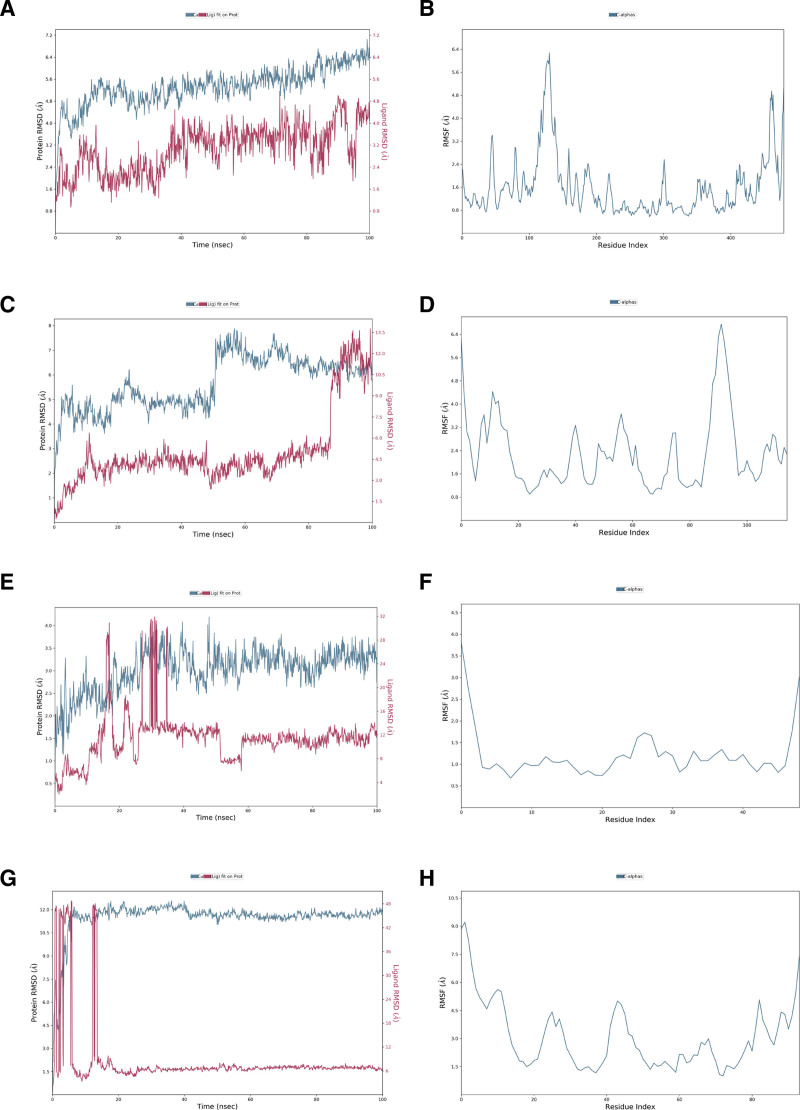
Molecular dynamics simulation of the complex of potential active ligands of Jiawei Shoutai Pill and key target proteins for treating DOR. DOR = diminished ovarian reserve.

To further validate these results, we conducted molecular dynamics simulations of 4 complexes: kaempferol–AKT1, luteolin–TP53, quercetin–IL-6, and quercetin–TNF. Figure 9A shows the root mean squared deviation (RMSD) trajectories of the molecular dynamics simulations of the protein (left *y*-axis) and ligand (right *y*-axis) in the kaempferol–AKT1 complex. The RMSD trajectory of AKT1 suggested that the protein underwent a large conformational change throughout the simulation process; however, it stabilized at approximately 5.2 Å after 40 ns, suggesting that the protein conformation was relatively stable at this stage. The RMSD trajectory of the ligand remained stable after 40 ns and within the range of 2.4–3.6 Å, indicating that the ligand could combine stably with the protein at this stage. The RMSF (root mean squared fluctuation) map of the protein in the kaempferol–AKT1 complex (Fig. 9B) showed that most of the amino acid residues of AKT1 fluctuated less after binding to kaempferol, indicating stable binding of the protein to the small molecule.

Figure 9C shows the RMSD trajectories of the molecular dynamics simulations of the protein (left *y*-axis) and ligand (right *y*-axis) in the luteolin–TP53 complex. The trajectory of TP53 fluctuated greatly at 50 ns, while the trajectory of the ligand fluctuated greatly at 50 ns. Similarly, the RMSF map of the protein in the luteolin–TP53 complex (Fig. 9D) showed that most amino acid residues fluctuated greatly.

Figure 9E shows the RMSD trajectories of the molecular dynamics simulations of the protein (left *y*-axis) and ligand (right *y*-axis) in the quercetin–IL-6 complex. The RMSD values of protein and ligand fluctuate between 2.7–3.7 and 1.3–1.7 Å, respectively. Although the ligand RMSD value fluctuated greatly between 20 and 30 ns, its amplitude was still within 3.0 Å. In the quercetin–IL-6 complex, the RMSF trajectory of IL-6 (Fig. 9F) also suggested that the complex was relatively stable.

Figure 9G shows the RMSD trajectories of the molecular dynamics simulations of the protein (left *y*-axis) and ligand (right *y*-axis) in the quercetin–TNF complex. Although the RMSD values of the protein and ligand fluctuated greatly in the early stage, the protein RMSD remained stable between 11.0 and 12.0 Å after 10 ns, and the ligand RMSD remained stable around 6 to 7 Å after 17 ns, indicating that the quercetin–TNF complex had good stability. The protein RMSF map (Fig. 9H) showed that TNF amino acid residues fluctuated greatly, and its binding with quercetin was more flexible.

The above molecular dynamics results showed that Quercetin binding to the corresponding proteins was more stable than that of kaempferol or luteolin, and could form stable complexes with IL-6 or TNF, which may be a pathway for Jiawei Shoutai Pill to treat DOR.

## 4. Discussion

The main treatment for infertility caused by decreased ovarian reserve function is assisted reproduction, but it is relatively more likely to occur in the process of in vitro fertilization-embryo transfer (IVF-ET) in the process of exogenous gonadotropin and inadequate oocyte retrieval, resulting in the cancelation of the cycle or adverse pregnancy outcomes.^[10]^ Therefore, researchers are constantly exploring effective schemes to improve the success rate of IVF-ET in patients with DOR, including the selection of superovulation schemes and pretreatment measures before the IVF-ET cycle.^[11]^ Each scheme has its own advantages and disadvantages. Choosing an appropriate individualized ovulation induction scheme for DOR patients and obtaining a higher success rate of IVF-ET.^[12]^ Traditional Chinese medicine plays an important role in assisted reproduction, which can improve the quality of eggs and endometrial receptivity to a certain extent through multiple targets and pathways, and further improve the success rate of IVF-ET.

In this study, the mechanism of Jiawei Shoutai Pill in the treatment of DOR was preliminarily studied using network pharmacology and molecular docking. The results identified 23 effective chemical components, 149 potential targets, and 170 related signal pathways. The top 4 chemical components were quercetin, luteolin, kaempferol, and naringenin. Yu et al^[13]^ showed that quercetin could protect fertilized eggs from H_2_O_2_-induced oxidative damage in mice by reducing reactive oxygen species, maintaining mitochondrial function, and regulating total antioxidant capacity and enzyme antioxidant activity to maintain the redox environment of cells. Huang et al^[14]^ found in an animal experiment that luteolin could enhance the antioxidant response by restoring the protein and mRNA expression of Nrf2 and downstream genes (such as Hmox1 and Nqo1) in rats, thereby protecting ovarian function. Yao et al^[15]^ showed that kaempferol could alleviate aging of porcine oocytes by reducing oxidative stress and improving mitochondrial function. Rumaisa et al^[16]^ found that naringenin treatment restored the expression levels of phosphorylated AKT, CYP17A1, CYP19A1, and 3β-HSD2 in human ovarian cells, improved ovulation potential, and reduced cystic follicle and androgen levels. Combined with the results of previous studies and ours, we speculate that the 4 chemical components of Jiawei Shoutai Pill play a key role in the treatment of DOR, which is worthy of further research and discussion.

The results of the protein interaction analysis showed that the top 5 targets were AKT1, TNF, IL-6, IL-1β, and TP53. AKT1 is 1 of 3 closely related serine/threonine protein kinases^[17]^ called AKT kinase, whose function can regulate many processes, such as metabolism, proliferation, cell survival, growth, and angiogenesis.^[18,19]^ PI3K/AKT is an important signaling pathway for regulating cell proliferation, apoptosis, and the cell cycle,^[20]^ which can up-regulate cell proliferation and inhibit apoptosis. Molecular experiments,^[21]^ shown that activation of the PI3K/AKT pathway can inhibit the release of cytochrome C to inhibit the apoptosis of granulosa cells, thereby improving ovarian function. However, lipopolysaccharide can reduce the expression of PI3K and AKT, increase the number of atretic follicles, reduce the number of growing follicles and luteum, and inhibit the production of estrogen.^[22]^ TNF is a substance that can cause hemorrhagic necrosis in a variety of tumors, mainly produced by activated macrophages, NK cells, and T lymphocytes. Among them, TNF-α is a cytokine that participates in the development, proliferation and apoptosis of ovarian follicular cells in domestic mammals^[23]^; IL-1β and IL-6 are important members of the cytokine network and play a central role in the acute inflammatory response. Studies have shown that by down-regulating the expression levels of TNF-α, IL-1β, and IL-6, cell viability can be significantly improved, and the apoptosis rate of ovarian granulosa cells can be significantly reduced.^[24,25]^ TP53 is a tumor suppressor gene that has been confirmed as an upstream gene of the monocyte-derived steroidogenic factor. Moreover, the increase in TP53 in ovarian granulosa cells inhibits the function of some granulosa cells by stimulating apoptosis signal-regulated kinase, blocking the maturation of adjacent oocytes^[26]^ and affecting ovulation and fertilization.^[27]^ Based on the molecular docking results of our team, it is speculated that luteolin monomer may positively intervene in granulosa cell expression by inhibiting TP53 expression to induce oocyte development, thereby improving ovarian function.

## 5. Conclusion

The pathogenesis of decreased ovarian reserve is not clear; therefore, although most fertility problems in patients with decreased ovarian reserve can be solved by symptomatic treatment, it still seriously affects women’s long-term health. Therefore, this study aimed to investigate the possible etiology of decreased ovarian reserve and explore the pharmacological mechanism between the Jiawei Shoutai Pill and DOR. We screened out the active compounds and corresponding gene targets of the Jiawei Shoutai Pill and further explored the pharmacological mechanism of them on DOR using a network pharmacology method. Our results provide 3 research directions for its therapeutic application: the PI3K–AKT signaling pathway, the TNF signaling pathway, and lipopolysaccharide metabolism. Our results also showed that the 3 main active compounds of Jiawei Shoutai Pill, quercetin, kaempferol, and luteolin, have potential pharmacological effects on DOR, and quercetin has a higher stability in binding to the target protein. Future studies should aim to verify the effect of quercetin on DOR to clarify its underlying mechanisms, and future studies should also focus on the lipid metabolism of DOR patients. In addition, the results showed that the same compound in the Jiawei Shoutai Pill could regulate multiple targets, and the same targets could intervene in multiple biological processes and pathways, which reflected the characteristics of Jiawei Shoutai Pill with multipathway and multitarget combinations.

This study integrates traditional Chinese pharmacology, high-throughput bioinformatics, and advanced data analysis software to conduct a preliminary exploration of the therapeutic mechanisms of Jiawei Shoutai Pill in treating DOR at the theoretical level. It uncovers the potential mechanisms of action, lays a scientific foundation for the study of the pharmacological substances and therapeutic efficacy, and provides guidance for future research directions.

However, the study is still subject to influences from factors such as the content of ingredients, their interactions, and the metabolic processes within the body. Future research will need to delve deeper into the material basis and pharmacological mechanisms underlying the efficacy of Jiawei Shoutai Pill in treating diminished ovarian reserve, so as to enhance the rationality and scientific basis of its clinical application.

## Author contributions

**Conceptualization:** Ying Zhao.

**Data curation:** Xiaoyue Lyu, Chun Shen.

**Funding acquisition:** Ying Zhao.

**Investigation:** Yumin Fang.

**Methodology:** Xiaoyue Lyu.

**Project administration:** Xiaoyue Lyu.

**Resources:** Ying Zhao.

**Software:** Ying Zhao.

**Visualization:** Yumin Fang.

**Writing – original draft:** Xiaoyue Lyu, Chun Shen.

**Writing – review & editing:** Xiaoyue Lyu.
